# Seasonal dynamics and functional diversity of frugivorous bird-plant interaction network in the karst habitat

**DOI:** 10.3389/fpls.2026.1798322

**Published:** 2026-03-24

**Authors:** Guohai Wang, Yanru Wang, Huangmin Zhang, Mengjie Nong, Lijuan Wei, Jifeng Long, Jianfeng Wu, Chiyung Jim, Qihai Zhou

**Affiliations:** 1 College of Agriculture and Biology, Guangxi Minzu Normal University, Chongzuo, Guangxi, China; 2 Key Laboratory of Ecology of Rare and Endangered Species and Environmental Protection, Ministry of Education, Guangxi Key Laboratory of Rare and Endangered Animal Ecology, Guangxi Normal University, Guilin, Guangxi, China; 3 College of Mathematics and Computer Science, Guangxi Minzu Normal University, Chongzuo, Guangxi, China; 4 Administrative Center of Nonggang National Nature Reserve, Longzhou, Guangxi, China; 5 Department of Social Sciences and Policy Studies, Education University of Hong Kong, Tai Po, Hong Kong SAR, China

**Keywords:** bird and fruit trait, frugivorous bird, fruiting plant, functional diversity, interaction network, karst habitat

## Abstract

**Introduction:**

The karst region of southwest China is characterized by fragmented habitats with high plant diversity but low resource availability, and its strong topographic and edaphic heterogeneity drives spatial variation in resource distribution, ultimately structuring frugivorous bird-plant interaction networks.

**Methods:**

We established six 2-3 km transects in a nature reserve in southern China. We conducted year-round observations of interactions between plants and frugivorous birds, subsequently using metrics to examine network structure and the functional trait of plants and birds shaping their network roles.

**Results:**

We recorded 196 unique links and 3,327 interactions events involving 19 frugivorous bird and 23 fruiting plant species. Compared with the null-model network (N=1000), the observed network exhibited lower connectance (C_z-score_=-23.845) and weighted nestedness (*w*NODF_z-score_=-9.215), as well as higher specialization (H2´_z-score_=55.042) and higher modularity (Q_z-score_=20.635). Connectance and weighted nestedness were higher during the dry season, whereas modularity and specialization peaked in the rainy season. Plants had higher functional richness and evenness, and birds had higher functional redundancy across periods. Plants experienced a notable increase in functional richness and dispersion from the dry to the rainy seasons, whereas birds had more stable functional diversity. *Pycnonotus jocosus* and *P. sinensis* birds showed the highest species degree (both 23), closeness centrality (12.145 and 10.070, respectively), and betweenness centrality (0.434 and 0.176, respectively). *Ficus concinna* and *F. altissima* exhibited the highest species degree (both 16), and *F. concinna* and *F. religiosa* shared the highest closeness centrality (both 11.757). Bird body mass was the only trait with a significant negative impact on species degree (β=-1.280, P=0.014).

**Discussion:**

These findings highlight the seasonal variation in frugivorous bird-plant networks in the karst habitats and species traits affecting network functional roles, providing new insights into the mechanisms sustaining interaction networks in heterogeneous, resource-limited ecosystems.

## 1 Introduction

The interactions between frugivorous birds and fruit plants are the functional core of terrestrial ecosystems, mediating critical ecological processes such as seed dispersal, biodiversity persistence, nutrient cycling, plant population regeneration, and resilience to environmental fluctuations (Purificação et al., 2014; Vizentin-Bugoni et al., 2019). These interactions are not merely binary encounters but are embedded within complex, multi-species networks that structure communities and drive ecosystem functioning (Simmons et al., 2018; Plein et al., 2013). The stability and resilience of these interaction networks are thus essential for maintaining biodiversity, especially in the face of escalating anthropogenic pressures such as habitat fragmentation and climate change (González-Castro et al., 2015; Guimarães, 2020).

Interaction networks between frugivorous birds and plants often exhibit remarkable spatiotemporal dynamics and structural complexity, primarily driven by the spatiotemporal turnover of species and individuals (Moulatlet et al., 2023). The structural characteristics, stability, and ecological functions of these networks can be quantified by topological parameters (Li et al., 2020; Berón et al., 2025). For instance, connectance quantifies the number of interspecific interactions, modularity identifies the clustering patterns of species within communities, nestedness assesses the hierarchical organization of interactions, and specialization measures the specificity of species-specific associations (Fernández and Fontúrbel, 2021; Howes et al., 2023). These metrics provide key insights into network resilience and underlying ecological processes, thereby establishing a fundamental analytical framework that links macroscale network patterns to microscale driving mechanisms (Montoya-Arango et al., 2019; Pinto et al., 2021).

Trait-matching process between species’ morphological and behavioral characteristics is a pivotal determinant of frugivorous bird-plant interaction network structure (Teodosio-Faustino et al., 2021; Wang et al., 2023). Within this framework, functional diversity serves as a key link that quantifies the composition, abundance distribution, and value range of functional traits across species in a community (Albrecht et al., 2018). Also, it connects these matching processes to emergent network properties as they arise (Mariano-Neto and Santos, 2023). Plant traits (fruit size, fruiting phenology, nutritional content, and plant height) and bird traits (bill length, body mass, and foraging behavior) filter mutually compatible species into interactive partnerships, thereby directly shaping core dimensions of functional diversity (Kuebbing et al., 2018; Schleuning et al., 2015). For example, a bird’s gape width determines the size of fruits it can consume, a plant’s fruiting phenology controls the temporal window for interactions with dispersers, and fruit traits such as nutritional composition, size, availability, and color influence bird foraging decisions (Blendinger et al., 2015; Ordano et al., 2017). Collectively, these two-way mechanisms not only shape the complexity of interaction networks but also modulate their functional diversity by enhancing trait complementarity (Pena et al., 2023). Therefore, they collectively determine the distribution and expression of functional traits within the network and lay the foundation for quantifying network-level functional diversity metrics (Sebastián-González et al., 2017; Sobral et al., 2010).

The karst region in southwestern China constitutes a unique geological-ecological landscape characterized by highly exposed rocks, limited surface water, and sparsely and unevenly distributed soils (Chen et al., 2021). The topography and strong environmental filtering in this habitat altered the diversity and spatial distribution of frugivorous birds and plants, ultimately shaping the patterns and strength of interactions between them (Wang et al., 2025). These karst−specific ecological conditions accentuate trait−matching processes between birds and plants, thereby providing a natural experimental setting to examine empirically how trait−mediated assembly rules influence interaction network structure. Despite its ecological uniqueness, little research has been conducted on the intricate and complex interactions between frugivorous birds and plants in the karst habitat.

The Guangxi Nonggang National Nature Reserve in China’s northern tropical region safeguards the largest, most structurally intact, and most representative seasonal karst rainforest, supporting exceptional and unique biodiversity (Wang et al., 2014). Preliminary surveys have revealed that many bird species rely on fruits as a food resource during the fruiting period. We hypothesized that the network structure would exhibit seasonal differences and that the roles of different species in the interaction network would be affected by the interplays between bird and plant traits. Based on the premise of the existence of complex interaction networks between frugivorous birds and plants, we addressed three core research objectives: (1) analyze potential changes in the structural and functional diversity of frugivore bird-plant interaction network in the karst habitat from the dry to the rainy season; (2) identify the most important frugivore birds and plants in the interaction network; and (3) evaluate the effects of bird and plant species traits on their network functional roles.

## 2 Materials and methods

### 2.1 Study site

This study was conducted in Guangxi Nonggang National Nature Reserve (106°42′−107°4′E, 22°13′−22°39′N), southern Guangxi Province, China, which is located at the northern edge of the tropics. The reserve covers 10,080 ha and comprises three portions separated from each other by farmlands and villages: Nonggang (5,426 ha), Longhu (1,034 ha), and Longshan (3,949 ha). Our research site is in Nonggang (106°42′–107°00′E, 22°13′–22°33′N). It has an annual average temperature of 22 °C and annual rainfall of 1,150–1,550 mm, with 76% occurring in May to September. The climate has marked seasonality in temperature, rainfall, and plant resource availability. Based on average temperature and rainfall, the year is divided into the rainy season (April–September) and the dry season (October–March of the following year). The vegetation is a limestone seasonal rain forest, characterized by dense limestone hills and intervening flat lands at altitudes of 300–700 m above sea level (Wang et al., 2014).

### 2.2 Sampling of frugivore-plant interactions

**Figure 1:**
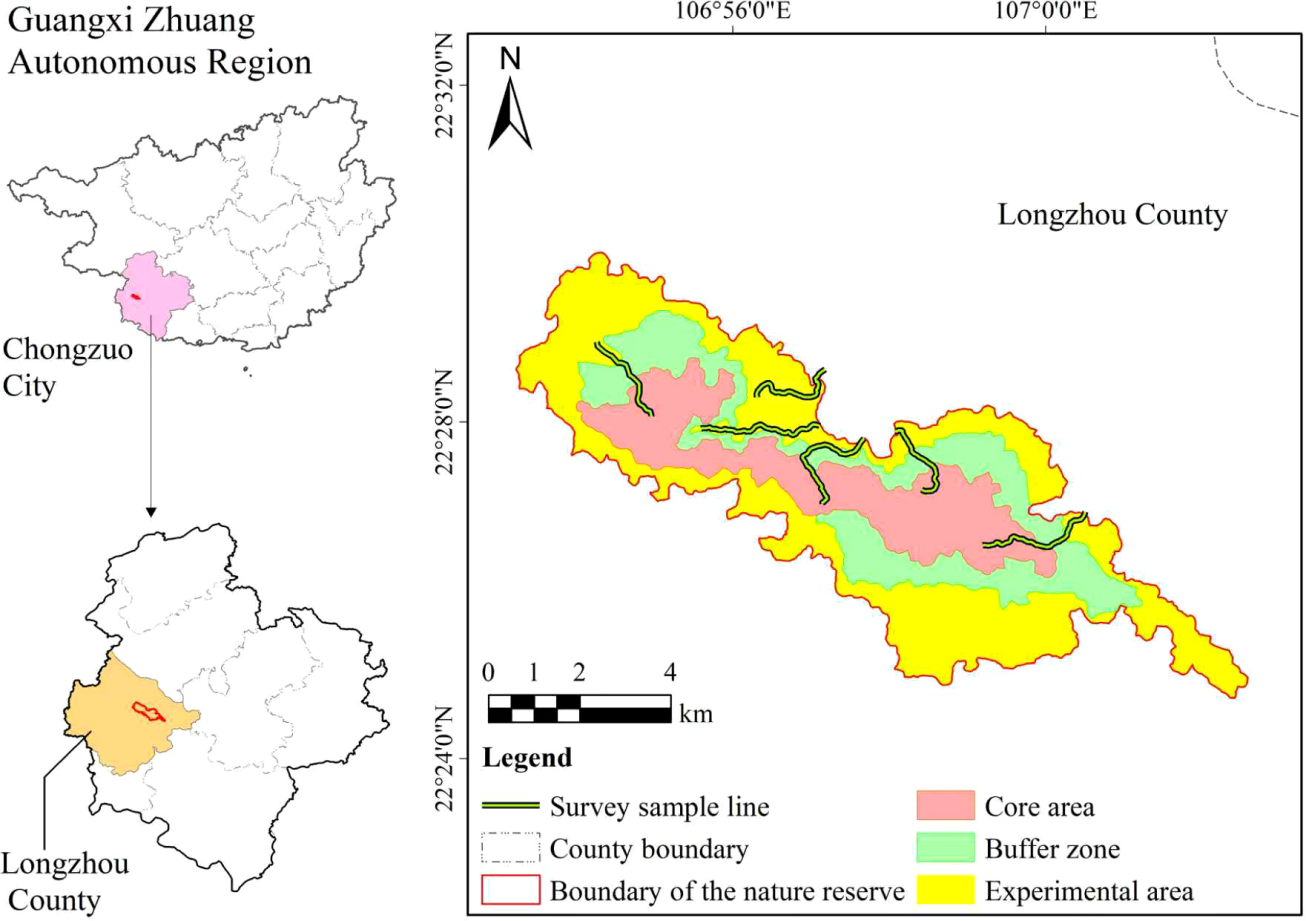
Distribution of the survey transects in Nonggang National Nature Reserve, southwest China.

Based on terrain conditions in the survey area, we established six transects, each 2−3 km each to observe the foraging behavior of frugivorous birds from January to December 2024 (**Figure 1**). On every sampling day, all frugivory events between birds and plants were observed during birds’ peak foraging periods (07:00−10:00 h and 15:00−18:00 h) with Safari l0×42 zoom binoculars. An interaction event was defined as a frugivorous bird flying to the crown of a fruit plant and eating at least one fruit before flying away from the plant (Wei et al., 2025). The investigator recorded the species of birds and plants, the foraging pattern, the number of fruits consumed, the number of birds per visit, and the foraging duration. If frugivorous birds visited the trees in conspecific flocks, the habits of one randomly selected individual bird were recorded and assumed to be characteristic of the entire feeding flock (Breitbach et al., 2010; Wang et al., 2023). All surveys were conducted on clear weather days, and the observation frequency was at least 8 days per transect per month.

### 2.3 Fruit and frugivore bird traits

To identify potential biological drivers of interaction roles within the network, we collected key morphological and nutritional traits for all bird and plant species. For plants, we measured fruit diameter (mm), fruit length (mm), fruit mass (g), fruit color, and fruit abundance index (FAI), along with nutritional content (sugar, fat, and protein). Fruit dimensions and mass were gauged using vernier calipers and an electronic balance, based on 30 fruits sampled from one to three individuals per species. Each fruiting plant species was assigned a value of ripe FAI according to an ordinal scale: 0=without fruits; 1 = 1−10 fruits; 2 = 11−100 fruits; 3 = 101−1,000 fruits; 4 = 1,001−10,000 fruits; and 5>10,000 fruits (Donoso et al., 2017; García, 2016). Sugar composition was analyzed via high-performance liquid chromatography (1260-IIPrime, Agilent Technologies), fat content was determined using Soxhlet extraction (SOX406, Haineng Future Technology Group), and protein content was estimated by quantifying nitrogen using the Automatic Kjeldahl apparatus (k1160, Haineng Future Technology Group) and applying a conversion factor of 6.25 (Maruyama et al., 2019).

For each bird species, we collected data on key morphological traits, including bill width (mm), body length (mm), body mass (g), wing length (mm), and tail length (mm), to assess their potential influence on network interactions. Trait data were primarily sourced from *A Field Guide to the Birds of China* (MacKinnon and Phillipps, 2000) and *A Handbook of the Birds of China* (Zhao, 2001).

### 2.4 Network-level analysis

To analyze interactions between plants and birds, a quantitative interaction matrix was constructed based on the number of interaction events. In this matrix, bird species are the columns, plants are the rows, and each cell value represents the number of interaction events between each bird-plant pair. We determined the sample coverage for bird and plant species, as well as for their interactions, using individual-based interpolation or extrapolation with sample completeness curves, implemented via the *i*NEXT function in the R package (Hsieh et al., 2016).

To quantify the structural properties of the interaction networks, we constructed three network subsets (overall, dry season, and rainy season). We calculated a set of widely used ecological metrics to capture their structural complexity and functional potential. These metrics included: (1) Connectance (*C*) measures the proportion of observed interactions relative to all possible interactions, computed as C=I/(A×B), where I is the number of recorded interactions, and A and B represent the total bird and plant species, respectively (Fernández and Fontúrbel, 2021); (2) Weighted nestedness (*w*NODF), based on overlap and decreasing fill, expresses the relationship between the average number of interactions per species and total species richness in the network (Costa et al., 2020); (3) Specialization (*H_2_*′) evaluates niche differentiation across the community by quantifying the overlap of frugivore species among different plants and the overlap of plant species among different frugivores (Blüthgen et al., 2006; Wang et al., 2023); (4) Modularity (*Q*) identifies tightly connected subgroups (modules) in which within-group interactions are stronger than those between groups, a pattern often associated with trait matching, functional groups, or habitat and dietary niches (Dormann and Strauss, 2014; Pinto et al., 2021). To evaluate whether the observed network metrics deviated from those produced by null models, we generated 1,000 randomized matrices using the r2dtable algorithm. Significance (p) was estimated as the number of times the null model produced a network with a score equal to or higher than that of the original matrix, divided by the total number of randomizations (Wang et al., 2025). All analyses were conducted using the “networklevel” function in version 4.3.3 of the R package “bipartite” (R Development Core Team, 2024), with statistical significance defined as p < 0.05.

To explore the variations in trait diversity and their relevance to network interactions, we analyzed four core functional diversity indices using the FD package (R v4.3.3), all weighted by species interaction frequency to better reflect the actual functional contributions of species within this interaction network. These indices were as follows: (1) Functional Richness (FRic) quantifies the volume of functional trait space occupied by interacting communities, with higher values indicating broader functional roles (e.g., diverse foraging strategies among birds, varied fruit resource types among plants) and greater interspecific differentiation (Villéger et al., 2008; Suárez-Castro et al., 2022); (2) Functional Evenness (FEve) refers to the regularity of species abundance distribution within functional trait space, with higher values reflecting both uniform species distribution in trait space and consistent allocation of functional roles and interaction intensities, thereby stabilizing resource-use dynamics in ecological communities (Mason et al., 2005); (3) Functional Dispersion (FDis) is defined as the mean distance of species to the trait space centroid (weighted by species abundance and interaction frequency), with higher values signifying greater functional differentiation, which enhances functional complementarity and the resilience of interaction processes (e.g., seed-dispersal effectiveness) amid season heterogeneity (Espinosa et al., 2025); and (4) Functional Redundancy (FRed): denotes the overlap of functional roles among species, buffering networks against partner loss and ensuring the persistence of interaction processes (Maure et al., 2018). A higher FRed value indicates that multiple species can perform the same interaction role (e.g., multiple bird species dispersing the same plant’s seeds), thereby enhancing network stability (Corral et al., 2021).

To control the effects of richness data and interaction heterogeneity, the network parameters (connectance, weighted nestedness, specialization, and modularity) were standardized by converting them to Z-scores prior to comparing the observed and null-model networks. The Z-score was calculated as Z=(Obs−Exp_null(1….n)_)/Sd_null_, where Obs is the observed value, and Exp_null(1…n)_ and Sd_null_ are the mean and standard deviation, respectively, derived from 1,000 null model randomizations (Wang et al., 2025).

### 2.5 Species-level metrics

In order to quantify the relative importance of the species within the network structure, three centrality measures were calculated based on the interaction matrix: (1) Species degree, defined as the number of interactions per species (Wang et al., 2025); (2) Closeness centrality, quantifies the proximity of a focal node (bird or plant) to all other nodes in the network, with higher values indicating more extensive interactions with partner species and greater potential to influence other network components (Ulrich and Peters, 2023; Hernández-Dávila et al., 2022); (3) Betweenness centrality, measures how frequently a species acts as an intermediary in paths connecting all pairs of nodes-reflecting its role as a connector between distinct network subcomponents, where species with positive values are critical for maintaining network cohesion (Emer et al., 2018; Li et al., 2020).

To evaluate the effects of bird and plant species traits on their network functional roles, we conducted the following analyses. Given the significant scale discrepancies across variables, we first subjected all data to log_10_ transformation to enhance the linearity of subsequent analyses. For the betweenness centrality variable, which contained zero values, we used the formula log_10_ (variable + 1) to avoid the undefined value of log_10_ (0). Subsequently, one-sample Kolmogorov-Smirnov tests were performed on the transformed data, confirming that all variables were normally distributed. Generalized linear models (GLMs; “lme4” package) with Gaussian error distribution were applied to estimate the effects of species traits on their network roles. Model averaging (dredge and model.avg functions in the “MuMIn” package) was then used to evaluate and synthesize the results, with model selection based on the Akaike Information Criterion (AIC). We set the transformed network parameters (species degree, closeness centrality, and betweenness centrality) as the response variable, with transformed bird and plant traits as explanatory variables. Bird traits included bill width, body length, body mass, wing length, and tail length. Plant traits included fruit diameter, fruit length, fruit mass, fruit color, fruit abundance index (FAI), and fruit nutrition (sugar, fat, and protein). The analyses were conducted using R version 4.3.3 (R Development Core Team, 2024). All tests were two-tailed, with a significance level of 0.05.

## 3 Results

### 3.1 Structure of frugivorous bird-plant interaction network

**Figure 2:**
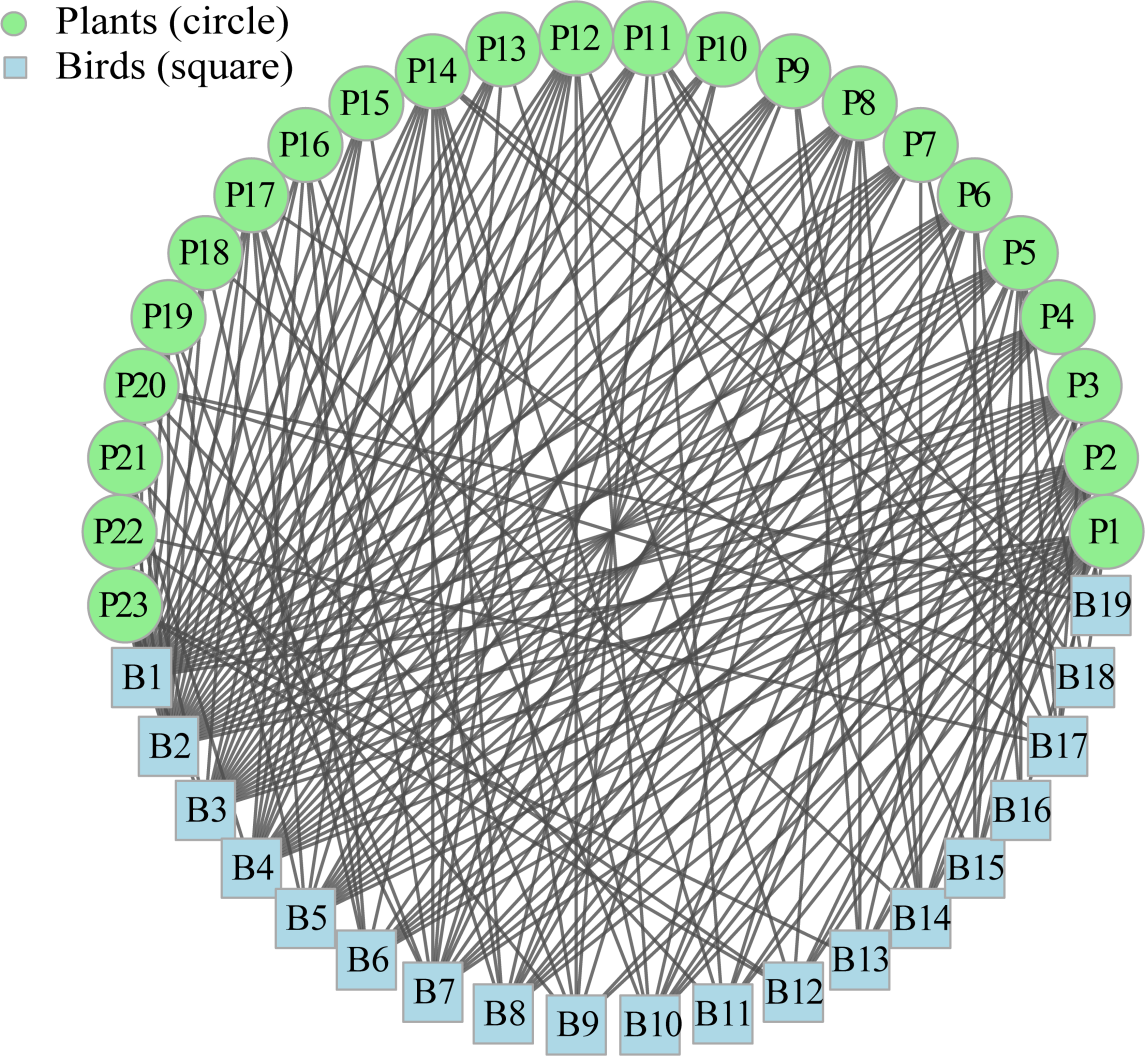
The complex frugivorous bird-plant interaction network in the karst habitat. B1 to B19 represent 19 species of frugivorous birds, and P1 to P23 represent 23 fruit plant species. See **Table 3** for corresponding species names.

The size of the interaction network was 19 bird species and 23 plant species, with a total of 3,327 interactions observed throughout the year (**Figure 2**). Each fruit plant species interacted with 8.52 ± 0.79 (Mean ± SD) birds, and each bird species interacted with 10.32 ± 1.39 fruit plant species. *Ficus concinna*, with 565 interactions (16.98% of the total), was the most consumed by birds. At the same time, *Pycnonotus jocosus*, the bird species most frequently recorded, accounted for 1,704 interactions (51.23% of the total) (**Figure 2**).

**Figure 3:**
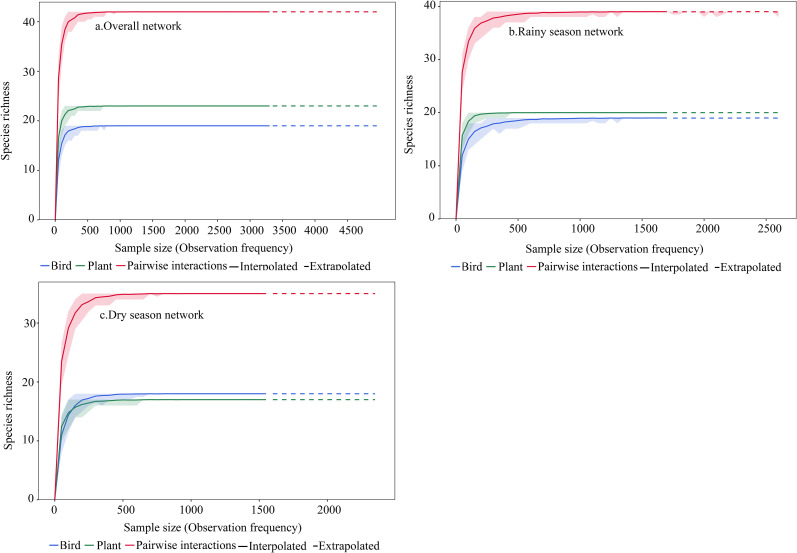
Sampling coverage of the frugivorous bird-plant interaction network in the karst habitat. Observed (interpolated, solid line) and simulated (extrapolated, dashed line) data for paired interactions (blue), plants (green), and birds (red) are well-represented by the sampling coverage across **(A)** overall network, **(B)** rainy season network, and **(C)** dry season network.

Sample coverage of frugivorous bird-fruit plant interactions reached 99.81% in the overall network (birds: 99.52%; plants: 99.61%), 99.91% in the rainy season (birds: 99.35%; plants: 99.25%), and 99.75% in the dry season (birds: 99.50%; plants: 99.50%), indicating sufficient sampling of interactions without seasonal bias in completeness (**Figures 3A–C**).

**Figure 4:**
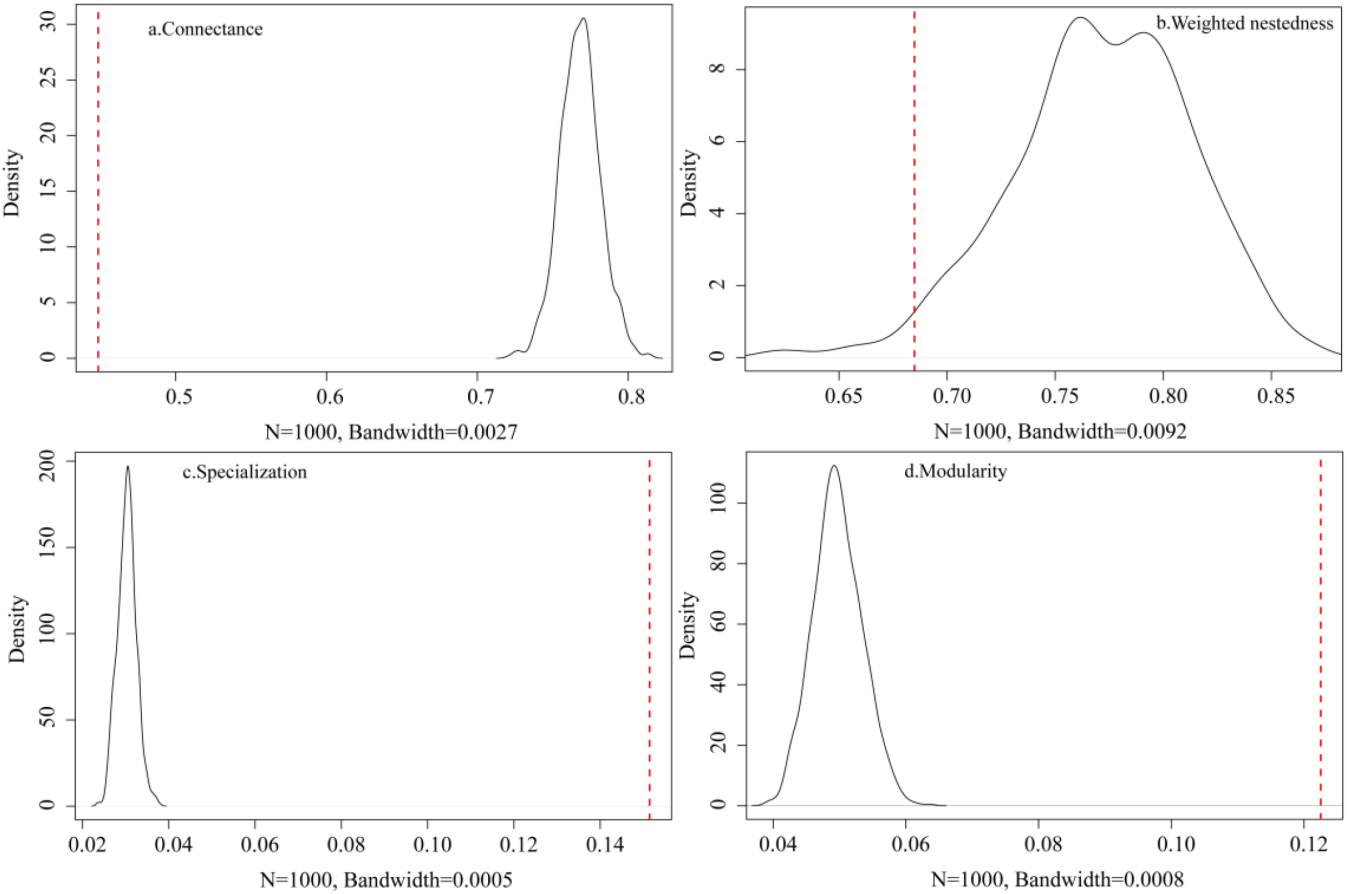
Comparison between the observed network and the networks generated by the null model. **(a)**: Connectance; **(b)**: Weighted nestedness; **(c)**: Specialization; **(d)**: Modularity. The red line represents the parameter values of the actual observed network matrix, and the black line represents the parameter values of the network randomly generated by the null model.

At the network level, the observed network structure showed a lower connectance (*C*_z-score_=-23.845; *P* < 0.001) and weighted nestedness (*w*NODF_z-score_=-9.215; *P* < 0.001), as well as high specialization (*H_2_*´_z-score_=55.042; *P* < 0.001) and modularity (*Q*_z-score_=20.635; *P* < 0.001) than the random networks produced by the null model (N = 1,000; **Table 1**; **Figure 4**). All metrics differed significantly in relation to the null model. Seasonally, the rainy and dry seasons accounted for 52.36% and 47.64% of annual network connections, respectively (**Table 1**). Connectance and weighted nestedness were higher during the dry season, whereas modularity and specialization peaked in the rainy season. Moreover, all metrics in both seasons also differed significantly from the null model.

**Table 1:** Metrics characterizing the frugivorous bird-plant interaction networks in the karst habitat.

Parameter	Overall	Rainy season	Dry season
Bir richness	19	19	18
Plant richness	23	20	17
Number of links	196	149	119
Interaction frequency	3,327	1,742	1,585
Connectance (*C*_z-score_)	-23.845**	-20.377**	-13.907**
Weighted nestedness (*w*NODF_z-score_)	-9.215**	-7.159**	-4.686**
Specialization (*H_2_*´_z-score_)	55.042**	38.210**	30.459**
Modularity (*Q*_z-score_)	20.635**	19.320**	13.871**

**p<0.01.

### 3.2 Changes in network functional diversity

Across all periods, plants exhibited higher functional richness and evenness than birds, indicating that they possessed more diverse, evenly distributed and distinct functional traits (**Table 2**). In contrast, birds showed higher functional redundancy, meaning that multiple species could fulfill similar ecological roles to support functional stability. Plant functional richness and dispersion rose notably from dry to rainy seasons, but bird indices like functional richness stayed stable. Both groups showed only minor fluctuations in functional evenness and redundancy across seasons. Overall, plants had higher functional richness and dispersion and stronger seasonal responsiveness (especially to the rainy season), while birds had higher functional redundancy and more stable functional diversity.

**Table 2:** Functional diversity parameters of the frugivorous bird-plant interaction networks in the karst habitat.

Variable	Overall		Dry season		Rainy season	
	Bird	Plant	Bird	Plant	Bird	Plant
Functional richness (FRic)	0.031	0.091	0.032	0.109	0.031	0.135
Functional evenness (FEve)	0.451	0.710	0.457	0.717	0.451	0.722
Functional dispersion (FDis)	14.375	22.214	15.629	24.030	17.246	26.870
0Functional redundancy (FRed)	0.733	0.563	0.728	0.549	0.733	0.580

### 3.3 Importance of frugivorous bird-plant interaction network

The species degree of birds in the observed overall network ranged from 3 to 23, reflecting a high variability in the number of interactions across species in the network; *Pycnonotus jocosus* and *P. sinensis* (both 23) had the highest species degree values overall, a pattern indicative of their generalist foraging strategy and broad direct interactions. These species also ranked highest in closeness centrality (12.145 and 10.070, respectively) and betweenness centrality (0.434 and 0.176, respectively), underscoring their role as key connectors that bridge otherwise separate modules within the network (**Table 3**).

**Table 3:** Species-level metrics for the overall frugivory bird-plant interaction network in the karst habitat.

Code	Species	Species degree	Closeness centrality	Betweenness centrality
Bird species				
B1	*Pycnonotus jocosus*	23	12.145	0.434
B2	*Pycnonotus sinensis*	23	10.070	0.176
B3	*Zosterops japonicus*	18	8.885	0.159
B4	*Pycnonotus aurigaster*	16	9.423	0.099
B5	*Phylloscopus inornatus*	13	9.872	0.023
B6	*Hemixos castanonotus*	10	8.281	0.004
B7	*Alophoixus pallidus*	14	8.360	0.061
B8	*Pycnonotus xanthorrhous*	11	8.832	0.024
B9	*Lonchura punctulata*	8	8.202	0.001
B10	*Rubigula flaviventris*	9	8.693	0.006
B11	*Copsychus saularis*	7	6.263	0.001
B12	*Urocissa xanthomelana*	7	6.104	0.002
B13	*Passer montanus*	6	5.464	0.024
B14	*Spizixos semitorques*	8	6.540	0.001
B15	*Spilopelia chinensis*	5	7.838	0.000
B16	*Streptopelia orientalis*	3	5.307	0.000
B17	*Parus major*	7	4.849	0.000
B18	*Lonchura striata*	5	6.856	0.000
B19	*Acridotheres cristatellus*	3	2.929	0.000
Plant species				
P1	*Ficus concinna*	16	11.757	0.000
P2	*Ficus altissima*	16	10.872	0.102
P3	*Bischofia javanica*	13	9.705	0.034
P4	*Melia azedarach*	9	10.652	0.085
P5	*Camphora officinarum*	13	7.811	0.008
P6	*Triadica sebifera*	11	8.397	0.028
P7	*Flueggea virosa*	9	9.185	0.056
P8	*Broussonetia papyrifera*	12	8.453	0.029
P9	*Ficus racemosa*	8	10.069	0.084
P10	*Ehretia tsangii*	5	5.194	0.004
P11	*Morus alba*	9	9.395	0.061
P12	*Ficus tinctoria*	10	8.475	0.018
P13	*Eriobotrya japonica*	6	6.971	0.043
P14	*Sageretia thea*	12	6.725	0.017
P15	*Phyllanthus reticulatus*	5	8.277	0.070
P16	*Ficus subulata*	6	9.999	0.108
P17	*Ficus glaberrima*	8	6.848	0.049
P18	*Ficus cardiophylla*	4	6.873	0.006
P19	*Maclura cochinchinensis*	4	8.894	0.034
P20	*Ficus religiosa*	6	11.757	0.034
P21	*Syzygium cumini*	5	9.873	0.026
P22	*Averrhoa carambola*	4	9.623	0.020
P23	*Clausena lansium*	5	9.935	0.000

Among plants, *Ficus concinna* and *Ficus altissima* showed the highest species degree (both 16), while *F. concinna* and *Ficus religiosa* shared the highest closeness centrality (both 11.757). Betweenness centrality values were generally low across plant species, with the highest recorded in *F. altissima* (0.102), and one species (*Clausena lansium*) registered a value of 0 (**Table 3**).

Body mass was the only bird trait that had a significant negative association with species degree (β=-1.280, P = 0.014; **Table 4**), indicating that birds with the largest mass had low species degree. By contrast, none of the plant traits exhibited statistically significant correlations with the evaluated network parameters (**Table 5**).

**Table 4:** Results of generalized linear modeling (GLM) evaluating the effects of bird traits on their network role.

Variable	Estimate	Standard error	*Z*-value	*P*-value
Species degree				
Intercept	-2.820	2.097	1.266	0.206
Body length	2.933	1.491	1.815	0.069
Body mass	-1.280	0.486	2.463	0.014*
Bill width	-0.408	0.710	0.529	0.597
Tail length	0.454	1.341	0.321	0.748
Wing length	0.064	1.791	0.033	0.974
Betweenness centrality				
Intercept	0.049	0.079	0.581	0.561
Body length	-0.022	0.054	0.377	0.706
Body mass	-0.015	0.022	0.610	0.542
Bill width	-0.035	0.064	0.505	0.614
Tail length	-0.014	0.048	0.276	0.783
Wing length	-0.033	0.054	0.560	0.575
Closeness centrality				
Intercept	0.710	1.245	0.558	0.557
Body length	0.149	1.627	0.090	0.929
Body mass	-0.430	0.265	1.530	0.126
Bill width	-0.340	0.402	0.786	0.432
Tail length	0.892	0.469	1.787	0.074
Wing length	-0.951	0.672	1.310	0.190

*p < 0.01.

**Table 5:** Results of generalized linear modeling (GLM) evaluating the effects of plant traits on their network role.

Variable	Estimate	Standard error	Z-value	P-value
Species degree				
Intercept	0.336	0.395	0.816	0.414
Fruit length	-0.288	0.313	0.874	0.382
Fruit diameter	0.277	0.257	1.028	0.304
Fruit mass	-0.088	0.074	1.121	0.262
Fruit color	0.209	0.164	1.198	0.231
FAI	0.924	0.529	1.641	0.101
Protein	0.184	0.211	0.814	0.415
Fat	0.158	0.127	1.164	0.244
Sugar	0.028	0.062	0.434	0.664
Betweenness centrality				
Intercept	0.020	0.013	1.438	0.150
Fruit length	0.015	0.017	0.863	0.388
Fruit diameter	-0.004	0.020	0.182	0.855
Fruit mass	0.002	0.005	0.396	0.692
Fruit color	-0.018	0.013	1.346	0.178
FAI	0.008	0.042	0.170	0.865
Protein	-0.010	0.017	0.575	0.566
Fat	-0.002	0.010	0.210	0.833
Sugar	0.003	0.004	0.777	0.437
Closeness centrality				
Intercept	0.751	0.050	15.010	<0.001
Fruit length	0.137	0.323	0.420	0.671
Fruit diameter	0.131	0.314	0.420	0.677
Fruit mass	-0.011	0.120	-0.090	0.925
Fruit color	0.084	0.320	0.260	0.793
FAI	0.177	1.096	0.160	0.872
Protein	-0.060	0.392	-0.150	0.878
Fat	-0.008	0.217	-0.040	0.970
Sugar	-0.009	0.097	-0.090	0.930

## 4 Discussion

### 4.1 Niche heterogeneity driving specialization and diversity

Our analysis demonstrates that the frugivorous bird-plant network in the karst habitat exhibits significantly lower connectance and weighted nestedness than predicted by the null model (**Table 1**; **Figure 4**). Consistent with previous research, connectance in observation networks is commonly lower than that in null models (Zhang et al., 2022; Wang et al., 2023). This is pattern probably due to the intrinsic mathematical properties of the null model, which generates more connected matrices than most observed networks (Costa et al., 2020). Beyond these methodological considerations, the observed structural pattern is also closely associated with the unique environmental characteristics of the karst habitat and with species adaptability traits. The fragmented terrain and poor soil fertility in the karst landscape limit the spatial overlap between fruit resources and bird foraging ranges (Wang et al., 2025). In addition, asynchronous fruiting phenology of plants and high dietary specialization of frugivorous birds jointly narrow the temporal window for interspecific interactions, thereby reducing network connectance (Ramos-Robles et al., 2016). Moreover, ecological niche differentiation weakens interspecific competition, driving the system to form specialized species matching rather than the subset-superset association required for a nested structure (García, 2016). Furthermore, the lack of dominant fruiting plant species in the karst habitat also reduces the asymmetric interaction strengths needed to form weighted nestedness (Vázquez et al., 2007), resulting in lower nestedness in the network.

Compared with previous studies conducted in the karst habitat (Wang et al., 2025), the interaction network in our study encompasses a higher diversity of both frugivorous bird and plant species and exhibits a more complex interaction structure (**Figure 1**). This difference is primarily attributable to the high habitat heterogeneity of Nonggang Nature Reserve, which in turn provides more diverse niches for both plants and birds. Concurrently, the reserve’s large spatial extent and strong inter-patch connectivity also facilitate the maintenance of stable populations (Ma et al., 2023). Furthermore, the favorable climatic conditions at the tropical fringe enhance the availability of fruit resources, while low levels of human disturbance help preserve habitat quality (Hu et al., 2023). Collectively, these factors sustain a richer regional species pool, directly reflected in the network’s higher species richness, broader functional diversity, and greater structural complexity.

The observed interaction network showed significantly higher specialization and modularity than the null model (**Table 1**; **Figure 4**), consistent with previous studies (Wang et al., 2023, 2025). This pattern can be explained by the high rock outcropping in the karst habitat, which leads to patchy distributions of soil and vegetation. Such spatial patchiness not only restricts the activity ranges of frugivorous birds and constrains the growth and distribution of fruiting plants but also reduces the likelihood of holistic interspecific interactions (Wang et al., 2025). A high degree of specialization across the network as a whole further supported the roles of environmental filtering and niche differentiation (Moreno-Opo et al., 2016). In landscapes with heterogeneity in resource availability, birds may concentrate on specific plant partners or fruit types (García et al., 2011). Although this specialization can improve foraging efficiency, it also increases ecological vulnerability, as specialized species rely more on specific interaction relationships and are therefore more prone to co-extinction in fragmented habitats (Tinoco et al., 2017).

### 4.2 Seasonal variations shaping network structure

Our results indicate that the structure of the frugivorous bird-plant interaction network exhibits significant seasonal variations, with higher connectance and weighted nestedness during the dry season (**Table 1**). This structural pattern is closely associated with seasonal shifts in climatic conditions, particularly lower rainfall and higher temperatures in the dry season, which reduce fruit resource availability and reshape the foraging strategies of frugivorous birds. These drier and warmer conditions markedly decrease the diversity and abundance of fruiting plants, leaving only drought-tolerant taxa productive, thereby altering birds’ foraging choices and interaction partners (Wei et al., 2025). Faced with this resource concentration rather than overall species scarcity, frugivorous birds shift their foraging niches and focus their foraging activity on these dominant plant species (Pizo et al., 2022). This behavioral shift increases the frequency of interspecific interactions and overall network connectance. Also, it fosters asymmetric interaction frameworks centered on dominant plants, thereby promoting the subset-superset associations that underpin high levels of weighted nestedness (Bimler et al., 2024).

The interaction network exhibits higher modularity and specialization during the rainy season (**Table 1**), a pattern primarily driven by increased rainfall and milder temperatures that greatly enhance fruit resource availability. These conditions support greater fruiting plant diversity and asynchronous fruit phenology, reducing interspecific competition for fruits. Consequently, some bird species revert to their specialized foraging preferences and further partition their foraging niches according to fruit traits and spatial strata (Schubert and Walters, 2022). This niche differentiation facilitates the formation of multiple tightly interconnected species modules, thereby enhancing the network’s overall modularity (Juliana et al., 2020). Concurrently, it strengthens the specificity of pairwise interactions between birds and plants, driving the network’s specialization to peak during the rainy season.

### 4.3 Plants and birds displaying different functional diversities

Our results indicate that plants exhibit higher functional richness, evenness, and dispersion, while birds show greater functional redundancy (**Table 2**). This divergence reflects the distinct ecological attributes of these two interacting partners. The higher functional diversity of plants stems from the diverse assemblage of fruit in the nature reserve, and their distinct traits (e.g., fruit morphology, nutritional composition, and ripening time) collectively enhance overall functional diversity (Fuzessy et al., 2022). In contrast, birds exhibit higher functional redundancy due to low dietary specialization in dominant frugivorous groups (e.g., *P. sinensis* and *P. jocosus*), with overlapping foraging behaviors and fruit preferences allowing these groups to fulfill similar ecological functions (Morán‐López et al., 2020). This combination enhances the resistance and resilience of the whole karst ecosystem. Diverse plant functional traits ensure a stable, continuous resource supply across seasons. In contrast, high functional redundancy in birds helps sustain key ecological processes such as seed dispersal under environmental fluctuations or species loss.

Plant functional diversity exhibits seasonal variation, whereas bird functional diversity remains relatively stable across seasons (**Table 2**). This result reflects the different adaptation strategies of the two network partners in response to environmental changes. Abundant precipitation and favorable temperatures during the rainy season prompt plants to adjust their fruit ripening phenology to coincide with the peak activity periods of birds (Schubert and Walters, 2022), thereby enhancing plant functional richness and functional dispersion (Silva et al., 2021). In contrast, birds rely on their high mobility to cope with seasonal changes in fruit resources through flexible behavioral strategies. For example, during dry-season food shortages, they can expand their foraging ranges or switch to non-fruit resources (Chishty et al., 2021). Both taxa show slight seasonal variations in functional evenness and redundancy, indicating that a stable core structure within the interaction network can maintain the stability of key ecological functions in a changing environment. Overall, these parameters reveal a fundamental divergence in the strategies of plants and birds in the karst for coping with seasonal changes in environmental conditions, with plants relying on trait plasticity and birds on behavioral flexibility.

### 4.4 Influence of species-level centrality measures

Network species-level parameters highlight the key role of species in maintaining the structural stability of the bird-plant interaction network (Zhang et al., 2022). *P. sinensis* and *P. jocosus* exhibit the highest species degree values, reflecting their broad generalist foraging strategies and the most extensive direct interactions with fruiting plants. With large population sizes and flexible dietary habits, they can exploit fruits from a wide range of plant taxa at different sizes and phenological stages (Wang et al., 2023). Through these extensive interaction patterns, they not only maximize their own resource acquisition efficiency, but also become the most frequent direct interaction partners for most local fruiting plant species (Wei et al., 2025). Moreover, they have the highest closeness centrality values (**Table 3**), indicating a unique capacity to rapidly propagate ecological effects across the entire frugivore bird-plant network. Closeness centrality quantifies the average shortest path length between a focal species and all other species in the network (Ulrich and Peters, 2023). Thus, high values indicate that a species is positioned at the center of the interaction network, with minimal topological distance to both other frugivorous birds and plants (Li et al., 2020). Through frequent foraging interactions, they can accelerate the transmission of ecological signals, such as seed-dispersal media and resource-availability cues across different components of the network (Hernández-Dávila et al., 2022). These processes ensure that localized changes, such as a sudden fruiting peak of a plant species, can quickly ripple through the entire system to benefit multiple dependent taxa (Morán‐López et al., 2020).

Furthermore, these two bird species also rank high in betweenness centrality (**Table 3**), further highlighting their irreplaceable role as critical connectors and stabilizers in the network. Betweenness centrality measures how frequently a node lies on the shortest paths between other node pairs in the network, and higher values indicate that these birds serve as important ecological bridges, effectively connecting otherwise isolated or weakly linked modules (Li et al., 2020). For example, they often mediate interactions between early and late-fruiting tree species or establish connections between plant patches in the canopy and shrub layers, and these modules would rarely be connected without these generalist foragers (Plein et al., 2013). By promoting cross- module connections, these birds significantly reduce network fragmentation, enhance seed dispersal efficiency and increase energy flow throughout the system, thereby improving the overall stability of the interaction network and its ability to resist disturbances such as seasonal fruit shortages or local vegetation decline (Martins et al., 2025).

In contrast, the centrality patterns of plant species reflect a resource-driven network structure formed by the adaptive traits of plant combinations in the karst habitat. *F. concinna* and *F. altissima* exhibit the highest degree of centrality (**Table 3**), which confirms their position as the main fruiting tree resources. Their extensive connections with various bird communities highlight their irreplaceable role in maintaining local bird diversity and the stability of interaction networks, especially during seasons of fruit scarcity (Ayat and Tata, 2015). Moreover, *F. concinna* and *F. religiosa* exhibit the highest closeness centrality (**Table 3**), reflecting their unique capacity to indirectly connect with many other fruiting plants via shared bird dispersers. For instance, birds that feed on *F. concinna* fruits may subsequently visit other plant species, thereby establishing indirect ecological connections and enhancing the structural cohesion of the network (Wei et al., 2025). This connectivity is especially critical for maintaining resource complementarity in the heterogeneous karst habitat, where fruit availability varies widely across space and time (Wang et al., 2025).

However, most plant species exhibit low betweenness centrality (**Table 3**), indicating that they do not function as the primary bridging species maintaining inter−patch connectivity within the network. Although plants can mediate inter−patch interactions and modulate their topological roles through phenological and morphological traits, including seed size and fruiting period length (Vidal et al., 2014), they lack the capacity to actively control or direct such inter−patch interactions. Their integration into the network depends on the foraging behavior of frugivorous birds (Silva et al., 2022). By foraging across the landscape, birds connect geographically isolated plant patches, determine which plant species form interspecific connections via selective behavior, modulate link strength based on visit frequency (Mueller et al., 2014), and thereby act as critical intermediaries that sustain the network’s overall structure and integrity (García, 2016).

### 4.5 The role of inherent bird and plant traits

Bird body mass showed a significant negative association with species degree (**Table 4**), consistent with previous studies (Wang et al., 2025). The differences result from variations in bird physiological traits, flight energetic costs, and foraging ecological niche partitioning (García et al., 2015). Larger species generally incur higher energetic costs during flight and exhibit lower maneuverability, restricting their foraging range to localized and resource−dense areas (Albrecht et al., 2018). Such morphological constraints lead larger birds to preferentially use resource-dense patches and nutrient-rich fruits, maximizing energy intake while minimizing flight costs (Dehling et al., 2016; González-Castro et al., 2015). As a result, their more selective foraging behavior and narrower foraging range reduce the number of interaction partners and thus lower their species degree in the network.

Plant traits did not significantly influence species roles in the interaction network (**Table 5**), contrasting with results from previous studies (Pigot et al., 2016). This discrepancy is likely attributable to the biological characteristics of frugivorous bird-plant interaction networks and the spatiotemporal variability of fruit resources in the karst habitat. The structure of the interaction network is primarily shaped by the foraging preferences, mobility and population dynamics of frugivorous birds (Pena et al., 2023). For example, generalist species such as *P. sinensis* and *P. jocosus*, which can simultaneously consume fruits with different morphological and phenological characteristics, weaken the association between specific traits such as fruit size, color and phenology and network parameters. Moreover, the patchy distribution of fruit resources across microhabitats leads to spatial variation in interaction frequencies, even among conspecific plants with fixed traits (Wang et al., 2025); simultaneously, seasonal fluctuations in fruit resources add a temporal dynamic to the bird-plant network, these effects obscure the association between the studied plant traits and network parameters (Pizo et al., 2022). Last but not least, network parameters represent an integrated outcome of synergistic trait interactions rather than a direct reflection of any single trait (Martins et al., 2025). This study primarily focused on the quantitative analysis of individual fruit traits. However, did not further explore the combined effects of trait syndromes composed of these traits, such as fruit size, nutritional traits, phenological traits, and dispersal patterns. Consequently, it may have overlooked the core drivers of the interaction network, which could explain the statistically non-significant relations.

### 4.6 Limitations of the study

This study demonstrates that frugivorous birds and fruit plants in the karst habitat can form complex interaction networks, providing new insights into the mechanisms that maintain these networks in heterogeneous, resource-limited ecosystems. However, as the data were derived solely from one year of bird foraging behavior, they cannot fully quantify and disentangle the effects of seasonal turnover in frugivorous bird assemblages and plant phenological dynamics on interaction network structure. Future research requires long-term monitoring to track seasonal and interannual variations in the interaction network, and to integrate bird foraging behavior traits with plant phenological rhythms to clarify the mechanisms by which bird activities sustain the functional stability of karst ecosystems.

## 5 Conclusion

Our research demonstrated that the frugivorous bird-plant interaction network in the karst habitat exhibits strong seasonal variation. Plants display high functional diversity, whereas birds exhibit high functional redundancy. A few generalist bird species and Ficus plants act as core hubs, and interaction patterns are mainly driven by bird body mass. In contrast, the effects of plant traits are masked by strong spatiotemporal heterogeneity. Network stability relies on core hub species, plant functional diversity, and bird behavioral flexibility. These findings provide new insights into maintaining the stability of frugivore bird-plant interaction networks in karst ecosystems and establish a critical theoretical foundation for the conservation of regional biodiversity.

## Data Availability

The datasets presented in this study can be found in online repositories. The names of the repository/repositories and accession number(s) can be found in the article/supplementary material.
